# Evaluating dose of cisplatin responsible for causing nephrotoxicity

**DOI:** 10.1371/journal.pone.0215757

**Published:** 2019-04-25

**Authors:** Kyouko Higuchi, Takashi Yanagawa

**Affiliations:** 1 Department of Biostatistics School of Medicine, Kurume University, Kurume, Fukuoka, Japan; 2 Department of Pharmacy, Kurume University Hospital, Kurume, Fukuoka, Japan; 3 The Biostatistics Center, Kurume University, Kurume, Fukuoka, Japan; Tokushima University Graduate School, JAPAN

## Abstract

Nephrotoxicity is a well-known side effect of cisplatin for cancer treatment. Various regimens have been developed to treat cancer based on the type and severity of the tumor. We focus on the docetaxel, cisplatin, and 5-fluorouracil regimen, which is called the TPF regimen, where the standard dose of cisplatin is 60 mg/m^2^. The aim of this study is to examine the relationship of the dosage of cisplatin that causes nephrotoxicity and back ground factors of patients using information about the dose of cisplatin actually administered to patients. It is shown that nephrotoxicity may be caused by a substantially smaller dosage than the standard dose of cisplatin in the TPF regimen, indicating the need for dose adjustment, taking into account the patient’s background factors in the treatment of a cancer.

## Introduction

Cisplatin is an anticancer agent administered to patients with various types of cancer. Nephrotoxicity is one of the well-known major side effects of cisplatin. The pathophysiological mechanisms of cisplatin nephrotoxicity involve proximal tubular injury, oxidative stress, inflammation, and vascular injury of the kidney. In the proximal tubular injury, several different mechanisms are involved; these include apoptosis, autophagy, dysregulation of cell-cycle proteins, activation of the mitogen-activated protein kinase (MAPK) signaling pathways, direct toxic effects on renal epithelial cells, DNA damage, and mitochondrial dysfunction. It has been reported that 20–30% of patients treated with cisplatin develop nephrotoxicity after 1 to 2 weeks of administration [1–4]. Although various approaches have been developed to prevent cisplatin nephrotoxicity, such as adequate hydration with saline and urinary output by diuretics [5–8], renal damage still occurs.

Currently, various regimens are administered for the treatment of a cancer, based on cancer type and the severity of the disease. We focus in this study on the docetaxel, cisplatin, and 5-fluorouracil regimen, which is called the TPF regimen, where the standard dose of cisplatin is 60 mg/m^2^. Medical doctors often skip or discontinue the administration of cisplatin according to the patient’s condition [9, 10], but treatment is generally administered based on specific regimens where the dose of cisplatin is fixed. We may obtain the record of actual dose of cisplatin administered to each patient from the electric medical record system in the university hospital. The aim of this study is to examine the relationship of the dosage of cisplatin and background factors of patients using information about the dose of cisplatin actually administered to patients. First, we identified background factors of patients that were related to nephrotoxicity based on 87 patients who received the TPF regimen between January 1, 2013 and December 31, 2013 at the Kurume University Hospital. The baseline serum creatinine, body mass index (BMI), administration of non-steroidal anti-inflammatory drugs (NSAIDs), administration of magnesium oxide (MgO), and dose of cisplatin were identified. Next, risk groups were constructed by combining categories of those identified factors, except dose of cisplatin, and the dose of cisplatin at which nephrotoxicity was estimated to develop was computed in each risk group. We found that nephrotoxicity could be caused by a substantially smaller dose of cisplatin than the fixed standard dose of cisplatin in the TPF regimen. For example, patients who belonged to the risk group characterized by a baseline serum creatinine level of 0.56 mg/mL, BMI of 22.27 kg/m^2^, and with administration of NSAIDs and without administration of MgO were estimated to develop nephrotoxicity at about 27 mg/m^2^ cisplatin, much less than the 60 mg/m^2^ prescribed in the TPF regimen.

This study was approved by the ethical committee of Kurume University (No.14078). All data were fully anonymized before we accessed them and the ethics committee waived off the requirement for informed consent because this was a retrospective study, which used data that were stored in the electronic medical record system. The data set is available upon request to the first author of this manuscript.

## Materials and methods

### Patients

Clinical data of 418 patients who underwent cisplatin-based chemotherapy between January 1, 2013 and December 31, 2013 at the Kurume University Hospital were reviewed. Patients 18 years of age or younger, treated with arterial injection of cisplatin, treated with cisplatin as a radiation sensitizer, or who had no clinical data essential for the assessment of renal function were excluded. Since the incidence of nephrotoxicity depends on the cisplatin regimen [11–13], we focused on patients who received the TPF regimen, which was the most frequent regimen in our study. Sixty-nine patients were finally included.

TPF regimen is an induction chemotherapy regimen that generally involves administration of a single fixed dose of cisplatin before a surgical procedure. If the dose is found to be effective, another dose of cisplatin is often administered before the surgical procedure to enhance the effect. Eighteen of the 69 patients received the second dose and the average interval between the first and second dose was 60.2 days. Since the interval is long and the study was conducted by a pre-post design, we did not discriminate between the first and second drug doses and treated the data as if they were obtained from 87 patients (Table 1).

**Table 1:** Regimens of cisplatin-based chemotherapy.

Regimen	N (%)
TPF: Docetaxel, cisplatin, 5-flurouracil	87 (16.4)
CDDP+PEM: Cisplatin, pemetrexed	68 (12.8)
GC: Gemcitabine, cisplatin	67 (12.6)
AP: Doxorubicin, cisplatin	48 (9.0)
CDDP+VNR: Cisplatin, vinorelbine	40 (7.5)
CDDP+S1+TRT: Oral S-1, cisplatin, thoracic radiation therapy	37 (7.0)
CDDP+CPT-11: Cisplatin, irinotecan for cervical cancer	31 (5.8)
DCF: Docetaxel, cisplatin, 5-flurouracil for gastric cancer	31 (5.8)
CDDP+CPT-11: Cisplatin, irinotecan for lung cancer	28 (5.3)
CDDP+S1: Oral S-1, cisplatin,	24 (4.5)
CDDP+VP-16: Cisplatin, etoposide	23 (4.3)
CDDP+DTX+TRT: Cisplatin, docetaxel, thoracic radiation therapy	14 (2.6)
CDDP+5FU+Cet: Cisplatin, 5-flurouracil, cetuximab	9 (1.7)
M-VAC: Methotrexate, vinblastine, doxorubicin, cisplatin	7 (1.3)
CDDP+CPT-11: Cisplatin, irinotecan for gastric cancer	6 (1.1)
SPT: Oral S-1, cisplatin, trastuzumab	6 (1.1)
CDDP: Cisplatin	4 (0.8)
CDDP+GEM: Gemcitabine, cisplatin	2 (0.4)
Total	532 (100)

### Nephrotoxicity evaluation

The ratio of serum creatinine after cisplatin administration with respect to baseline serum creatinine was used for evaluating nephrotoxicity according to previous studies [14–16]. Following the U.S. National Cancer Institute Common Terminology Criteria for Adverse Events version 4.0 [17], a ratio ≥ 1.5 was defined as nephrotoxicity. Baseline serum creatinine was denoted by pre_scr. Post-treatment serum creatinine was defined as the maximum value of the values of creatinine measured in blood samplings during the course of cisplatin chemotherapy and denoted by post_scr. The distribution of ratios of post_scr to pre_scr had a tail skewed to the right; logarithmic transformation was performed to approximate it to a normal distribution and was denoted by log_rate_scr. Nephrotoxicity was equivalent to log_rate_scr ≥ 0.4.

### Cisplatin dose

The actual dose of cisplatin administered to each of the 87 patients was extracted from the electronic medical record system of Kurume University Hospital. Dividing it by the body surface area of the patient, the dose of cisplatin in mg per body surface area (mg/m^2^) of each patient was established.

### Data

The following patient information was collected from the electronic medical record system: age, gender, height, weight, dose of cisplatin, type of cancer, stage of cancer, chemotherapy regimen, white blood cell count, platelet count, hemoglobin, serum creatinine, serum albumin, serum sodium, serum potassium, and serum chloride. The following variables that are potential risk factors of renal function were also considered: history of smoking, complications of cardiovascular disease, diabetes, hypertension, hyperlipidemia, hyperuricemia, and administration of NSAIDs, MgO, calcium channel blockers, renin-angiotensin system inhibitor, other antihypertensive agents, antibiotics, and contrast medium [14–16, 18–24].

### Statistical analysis

The endpoint of this study was log_rate_scr. Identification of risk factors related to cisplatin nephrotoxicity was carried out in the following two steps. First, univariate regression analysis was conducted using log_rate_scr as the response variable and the background factors of patients as explanatory variables; variables whose level of significance was less than 20% were selected as potential risk factors. Second, a multiple regression model was constructed by using log_rate_scr as the response variable and the potential risk factors selected in the first step as explanatory variables. Although cancer stage was not selected in the first step, we added it to the multiple regression model since it was considered clinically important. Explanatory variables in the model were selected by applying a stepwise regression technique with a 15% significance level. Selected variables were called risk factors. The dose of cisplatin causing nephrotoxicity was computed as follows. First, risk groups were established by combining categories of selected risk factors in the stepwise regression, and then the dose of cisplatin that attained log_rate_scr = 0.4 was computed in each risk group. Statistical analysis was performed with the use of SAS software version 9.4 (SAS Institute, Cary, NC, USA).

## Results

### Background factors of patients treated with the TPF regimen

The study patients were treated with 18 regimens and received 532 courses of cisplatin. Table 1 lists the regimens of cisplatin-based chemotherapy. The table shows that the most frequent regimen was the TPF regimen (N = 87). Table 2 shows the background of patients treated with the TPF regimen and the results of univariate analysis. All of the patients were treated during hospitalization. The mean (± standard deviation) patient age was 62.9 ± 8.3 years old, the mean BMI was 19.9 ± 2.8 kg/m^2^, and 87.4% of the patients were men. The most common diagnosis was head and neck cancer (92%), and the most common complication was hypertension (32.2%). Thirty patients were treated with NSAIDs for pain management (34.5%), and 58 patients had stage 4 disease (66.7%).

**Table 2:** Results of univariate analysis: Means, number, and p-values of background factors of patients treated with the TPF regimen (N = 87).

**Background factors**	**Mean ± SD**	**p-value**
Age (years)	62.9 ± 8.3	0.78
BMI (kg/m^2^)	19.9 ± 2.8	0.19
Cisplatin dose (mg/m^2^)	52.6 ± 8.2	0.02
Clinical data		
White blood cell (×10^3^/μL)	66.2 ± 30.1	0.03
Hemoglobin (g/dl)	13.0 ± 2.0	0.29
Platelet (×10^3^/μL)	24.3 ± 9.3	0.38
Creatinine (mg/dL)	0.7 ± 0.1	< 0.001
Albumin (g/dL)	3.8 ± 0.5	0.81
Sodium (mg/dL)	140.2 ± 2.2	0.34
Potassium (mg/dL)	4.1 ± 0.4	0.32
Chlorine (mg/dL)	103.2 ± 2.8	0.27
**Background factors**	**No. (%)**	**p-value**
Male gender	76 (87.4)	0.78
History of smoking	64 (74.4)	0.91
Diagnosis		
Diabetes mellitus	8 (9.2)	0.25
Hypertension	28 (32.2)	0.77
Hyperlipidemia	10 (11.5)	0.05
Hyperuricemia	2 (2.3)	0.04
Cardiovascular disease	12 (13.8)	0.84
Use of drug with nephrotoxicity		
NSAIDs	30 (34.5)	<0.01
Antibiotics	5 (5.8)	0.83
MgO	8 (9.2)	0.20
Contrast media	0 (0.0)	
Calcium channel blockers	24 (27.6)	0.46
RAS inhibitors	12 (13.8)	0.38
Other antihypertensive agents	3 (3.5)	0.91
Cancer type		
Head and neck	80 (92.0)	0.32
Esophageal	7 (8.0)	0.32
Cancer stage		
1 / 2 / 3 / 4	1(1.2) / 12(13.8) / 16(18.4) / 58(66.7)	0.23

TPF, docetaxel, cisplatin, and 5-fluorouracil; BMI, body mass index; NSAIDs, nonsteroidal anti-inflammatory drugs; MgO, magnesium oxide; RAS, renin-angiotensin system.

### Univariate analysis

The following eight potential risk factors were selected from the background factors in the univariate regression analysis: pre_scr (p < 0.001), NSAIDs (p < 0.01), cisplatin dose (p = 0.02), white blood cell count (p = 0.03), hyperuricemia (p = 0.04), hyperlipidemia (p = 0.05), BMI (p = 0.19), and MgO (p = 0.20). Tumor stage was not a significant risk factor (p = 0.23).

### Multivariable analysis

The risk factors selected in the stepwise regression analysis were pre_scr (p < 0.0001), BMI (p = 0.003), NSAIDs (p = 0.009), MgO (p = 0.039), and cisplatin dose (p = 0.019). Table 3 lists the regression coefficients, standard error, t-value, and p-value of the selected risk factors. The predictive model of log_rate_scr was established as follows:

log_rate_scr=-0.130-0.849*pre_scr+0.029*BMI+0.147*NSAIDs-0.190*MgO+0.008dose

where

$$\text{pre}\_\text{scr}=\left\{ \begin{array}{ll} 0.56 & \text{if}\;\text{pre}\_\text{scr}\;\text{is}\;\text{in}\;\text{the}\;\text{first}\;\text{quartile}(0-\text{25}\%)\\ 0.63 & \text{if}\;\text{pre}\_\text{scr}\;\text{is}\;\text{in}\;\text{the}\;\text{second}\;\text{quartile}\;\left(25–50\text{\%}\right)\\ 0.78 & \text{if}\;\text{pre}\_\text{scr}\;\text{is}\;\text{in}\;\text{the}\;\text{second}\;\text{quartile}\;\left(25–50\text{\%}\right)\\ 0.89 & \text{if}\;\text{pre}\_\text{scr}\;\text{is}\;\text{in}\;\text{the}\;\text{fourth}\;\text{quartile}\;\left(75–100\text{\%}\right) \end{array}\right.$$

$$\text{BMI}=\left\{ \begin{array}{ll} 22.27 & \text{if}\;\text{BMI}\;\text{is}\;\text{above}\;\text{the}\;\text{median}\\ 17.47 & \text{if}\;\text{BMI}\;\text{is}\;\text{below}\;\text{the}\;\text{median} \end{array}\right.$$

$$\text{NSAIDs}=\{\begin{array}{ll} 1 & \text{if}\;\text{NSAIDs}\;\text{were}\;\text{administered}\\ 0 & \text{if}\;\text{NSAIDs}\;\text{were}\;\text{not}\;\text{administered} \end{array}$$

$$\text{MgO}=\{\begin{array}{ll} 1 & \text{if}\;\text{MgO}\;\text{was}\;\text{administered}\\ 0 & \text{if}\;\text{MgO}\;\text{was}\;\text{not}\;\text{administered} \end{array}$$

**Table 3:** Results of multivariate analysis: The regression coefficients, standard error, t-value, and p-value of the selected risk factors.

Risk Factor	Coefficient	Standerd error	t-value	p-value
Intercept	-0.130	0.272	-0.48	0.633
Pre_scr^a^	-0.849	0.190	-4.48	< 0.0001
BMI^b^	0.029	0.010	3.03	0.003
NSAIDs^c^	0.147	0.055	2.69	0.009
MgO^d^	-0.190	0.091	-2.10	0.039
Cisplatin dose	0.008	0.003	2.39	0.019

^a^baseline serum creatinine,

^b^body mass index,

^c^non-steroidal anti-inflammatory drugs,

^d^magnesium oxide.

Note that the values of pre_scr and BMI shown above are the median in each class.

### Estimation of cisplatin dose necessary for nephrotoxicity using the predictive model

Table 4 lists the 32 risk groups that were constructed by combining categories of the risk factors in Table 3. Dose levels that attained log_rate_scr = 0.4 in each group were computed using the predictive model and are listed in the sixth column in the table. The "#" in the table shows the risk groups whose computed dose levels were less than 60 mg/m^2^, that is, the fixed dose level of the TPF regimen. In other words, patients in the risk groups with "#" could have a strong possibility of developing nephrotoxicity below the fixed dose of cisplatin in the TPF regimen.

**Table 4:** Dose of cisplatin estimated to cause nephrotoxicity in 32 risk groups.

Group	Pre_scr^a^(mg/dL)	BMI^b^ (kg/m^2^)	MgO^c^	NSAIDs^d^	Cisplatin dose estimated to cause nephrotoxicity (mg/m^2^)
1	**0.56** first quartile (0–25%)	**22.27** above the median	with	with	50.33^#^
2				without	68.70
3			without	with	26.58^#^
4				without	44.95^#^
5		**17.47** below the median	with	with	67.73
6				without	86.10
7			without	with	43.98^#^
8				without	62.35
9	**0.63** second quartile (25–50%)	**22.27** above the median	with	with	57.77^#^
10				without	76.14
11			without	with	34.02^#^
12				without	52.39^#^
13		**17.47** below the median	with	with	75.17
14				without	93.54
15			without	with	51.42^#^
16				without	69.79
17	**0.78** third quartile (50–75%)	**22.27** above the median	with	with	73.68
18				without	92.06
19			without	with	49.93^#^
20				without	68.31
21		**17.47** below the median	with	with	91.08
22				without	109.46
23			without	with	67.33
24				without	85.71
25	**0.89** fourth quartile (75–100%)	**22.27** above the median	with	with	84.83
26				without	103.21
27			without	with	61.08
28				without	79.46
29		**17.47** below the median	with	with	102.23
30				without	120.61
31			without	with	78.48
32				without	96.86

^a^baseline serum creatinine,

^b^body mass index,

^c^magnesium oxide,

^d^nonsteroidal anti-inflammatory drugs.

## Discussion

The negative correlation between pre_scr and log_rate_scr (coefficient, -0.849; p < 0.001) in the predictive model can be explained as follows. The patients in the present study were cancer patients; in particular, 66.7% of them had stage 4 disease. It is known that cancer patients with higher stage have less muscle mass [25–30]. Furthermore, there are several studies that have reported that reduced muscle mass was related to low serum creatinine [31, 32]. Thirty patients (34.5%) had creatinine levels below the lower limit in this study, and there was no patient who exceeded the upper limit of the serum creatinine level; therefore, the negative correlation could be reasonably interpreted as patients with less muscle mass having a higher possibility of developing nephrotoxicity. Note that patients with chronic kidney disease were not included in this study.

Administration of MgO was also negatively correlated with log_rate_scr (coefficient, -0.190; p = 0.039). This is reasonable when we recall that magnesium injection has been recommended in recent years as a supportive treatment for renal protection in patients receiving cisplatin [22, 33, 34]; the magnesium injection was not included in the TPF regimen in 2013, the time the study data were collected.

The administration of NSAIDs as concomitant medication was shown to increase log_rate_scr. This is unsurprising, as NSAIDs are known to cause nephrotoxicity [16, 22, 35].

There are no previous studies that report BMI to be associated with cisplatin nephrotoxicity. However, it is well known that physiological functions greatly affect drug absorption, metabolism, and excretion, and correlate with body surface area [36]. Furthermore, as body surface area is correlated with BMI (Pearson’s correlation coefficient: 0.58), the association would be natural.

The negative coefficients of pre_scr and MgO in the predictive model indicates that patients who have less muscle mass and are not administered MgO could have an elevated possibility of developing nephrotoxicity at doses of cisplatin lower than the fixed standard dose. In contrast, the positive coefficients of NSAIDs and BMI in the predictive model indicate that patients who are not administered NSAIDs and those with a BMI below the median could have the possibility of developing no nephrotoxicity at the standard dose. Precise values of the cisplatin dose required for the development of nephrotoxicity in risk groups constructed by combining the categories pre_scr, MgO, NSAIDs, and BMI are shown in Table 4. The variation of doses that could lead to development of nephrotoxicity in risk groups is large, from 26.58 mg/m^2^ to 120.61 mg/m^2^, with a median of 71.74 mg/m^2^, but the lower values were concentrated mostly in patients with pre_scr in the first and second quartiles and who were administered NSAIDs.

In summary, the findings in this paper indicate the importance of adjusting the dose of cisplatin administered to cancer patients, taking into account their background factors. The findings should be an important message to the medical society when treating patients with cisplatin. We suggest adjusting the dose of cisplatin and providing support care for nephrotoxicity in each regimen, taking into account background factors of patients. If the dose of cisplatin, when reduced by the background factors is found to be weak on clinical assessment, then, it might be better to encourage the patients to seek alternative treatments, such as non-cisplatin regimen.

The sample size in this study is not large, mainly because we concentrated on patients receiving the TPF regimen. However, by doing so, the schedule of administration of anticancer drugs, the amount of hydration, and the type of diuretic and antiemetic drugs were all unified. Within the unified form, we could get reliable real-world background data of patients, as well as reliable clinical dose levels actually administered to patients. These data enabled us to identify risk factors, construct risk groups by combining categories of identified risk factors, and assess dose levels that may cause nephrotoxicity in each risk group. We hope this research is useful to decrease the incidence of cisplatin-induced nephrotoxicity in cisplatin treatment.

## Supporting information

S1 Table The background factors of patients treated with the TPF regimen. (PDF)
