# Supplementary material for: Evaluating dose of cisplatin responsible for causing nephrotoxicity
Source: PLoS One. 2019 Apr 25;14(4):e0215757. doi: 10.1371/journal.pone.0215757 (PMC6483206; doi:10.1371/journal.pone.0215757)
Supplement: S1 Table — (PDF) [file pone.0215757.s001.pdf]

S1 Table. The background factors of patients treated with the TPF regimen.

No.	Age	BMI	Dose	White blood cell	Hemoglobin	Platelet	Creatinine	Albumin	Male gender	History of smoking	Diabetes mellitus	Hypertension	Hyperlipidemia	Hyperuricemia	Cardiovascular disease	NSAIDs	Antibiotics	MgO	Contrast media	Calcium channel	RAS inhibitors	Other antihypertensive	Cancer type	Cancer stage	Body surface area
1	68	22.56	58.14	70	13.9	32.8	0.66	3.15	1	0	1	0	0	0	0	1	0	0	0	0	0	0	head and neck	4	1.72
2	79	18.61	38.22	40	13.0	18.2	0.76	3.74	1	0	0	1	0	0	0	0	0	0	0	1	1	0	head and neck	2	1.57
3	58	21.66	58.48	41	13.8	25.0	0.69	4.61	1	1	0	0	0	0	1	0	0	0	0	0	0	0	head and neck	3	1.71
4	66	20.63	46.30	50	14.2	6.1	0.67	3.89	1	1	1	1	0	0	0	0	0	0	0	0	1	0	esophageal	4	1.62
5	59	20.00	60.00	118	14.2	55.3	0.63	3.26	1	1	0	0	0	0	0	1	0	0	0	0	0	0	head and neck	4	1.5
6	67	17.30	60.40	93	15.2	26.3	0.46	4.71	1	1	0	0	0	0	0	0	0	0	0	0	0	0	head and neck	4	1.49
7	64	17.26	50.00	62	12.8	24.8	0.58	3.73	0	1	0	0	0	0	0	1	0	0	0	0	0	0	head and neck	4	1.4
8	64	16.70	35.97	70	11.0	25.4	0.58	3.3	0	1	0	0	0	0	0	0	0	0	0	0	0	0	head and neck	4	1.39
9	67	20.06	62.50	59	11.7	20.9	0.70	4.15	1	1	0	0	1	0	1	1	0	0	0	1	1	0	head and neck	3	1.6
10	67	19.28	60.51	65	9.0	29.9	0.70	3.89	1	1	0	0	1	0	1	1	0	0	0	1	1	0	head and neck	3	1.57
11	62	21.91	59.87	34	14.0	18.4	0.60	3.66	0	0	0	0	0	0	0	0	0	0	0	0	0	0	head and neck	2	1.57
12	63	22.94	44.03	45	12.8	19.8	0.58	3.97	0	0	0	0	0	0	0	0	1	1	0	0	0	0	head and neck	2	1.59
13	63	20.10	47.22	47	13.6	20.5	0.88	4.22	1	1	0	0	0	0	0	1	0	0	0	0	0	0	head and neck	2	1.8
14	63	21.72	47.77	71	13.0	17.9	0.56	4.4	1	0	1	1	1	0	1	1	0	0	0	1	0	0	head and neck	2	1.57
15	63	22.42	47.99	78	12.8	20.1	0.84	4.22	1	0	1	1	1	0	1	1	0	0	0	1	0	0	head and neck	2	1.59
16	78	15.80	35.71	72	11.8	23.9	0.76	3.39	1	1	0	0	0	0	0	1	0	0	0	0	0	0	head and neck	4	1.54
17	63	19.14	52.98	46	12.1	24.9	0.63	3.33	1	1	0	0	0	0	0	0	0	0	0	0	0	0	head and neck	4	1.51
18	70	23.81	35.84	71	11.6	15.9	0.81	4.03	1	1	0	0	0	0	0	1	0	0	0	1	1	0	head and neck	4	1.73
19	51	18.42	58.44	165	12.9	36.3	0.50	3.57	1	1	0	0	0	0	0	0	0	0	0	0	0	0	head and neck	4	1.54
20	51	17.67	59.60	197	11.6	40.9	0.64	3.2	1	1	0	0	0	0	0	1	0	0	0	0	0	0	head and neck	4	1.51
21	65	15.23	57.14	31	13.7	17.1	0.63	3.72	1	1	0	0	0	0	0	0	0	1	0	0	0	0	head and neck	4	1.4
22	65	16.24	59.03	39	13.5	17.6	0.59	4.16	1	1	0	0	0	0	0	0	0	0	0	0	0	0	head and neck	4	1.44
23	65	15.69	46.10	86	13.9	16.7	0.69	4.05	1	1	0	0	0	0	0	0	0	0	0	0	0	0	head and neck	4	1.41
24	66	15.56	46.10	42	13.2	18.2	0.61	4.09	1	1	0	0	0	0	0	0	0	0	0	0	0	0	head and neck	4	1.41
25	54	17.38	49.02	107	15.5	33.3	0.57	4.32	1	1	0	0	0	0	0	0	0	0	0	0	0	0	head and neck	3	1.53
26	58	21.30	59.51	45	13.6	27.6	0.60	3.21	1	0	0	0	0	0	0	0	0	1	0	0	0	0	head and neck	1	1.63
27	78	27.05	34.88	56	14.3	12.4	0.93	3.75	1	1	0	1	1	0	1	0	0	0	0	0	0	0	head and neck	2	1.72
28	62	17.69	58.82	68	14.6	16.4	0.84	4.22	1	1	0	1	0	0	0	0	0	0	0	0	0	0	head and neck	3	1.53
29	63	24.58	47.87	57	13.2	21.3	0.80	3.68	1	0	0	1	0	0	0	0	0	0	0	1	0	0	head and neck	4	1.88
30	62	22.89	45.98	67	14.1	24.5	0.77	3.8	1	0	0	1	0	0	0	0	0	0	0	1	0	0	head and neck	3	1.74
31	68	18.97	60.13	68	15.2	20.4	0.95	3.96	1	0	0	0	0	0	0	0	0	0	0	0	0	0	head and neck	3	1.58
32	41	16.73	60.13	52	12.2	23.3	0.80	4.49	1	1	0	0	0	0	0	0	0	0	0	0	0	0	head and neck	4	1.58
33	49	20.76	46.30	56	14.8	23.1	0.85	4.09	1	1	0	0	0	0	0	0	0	0	0	0	0	0	head and neck	2	1.62
34	73	21.11	47.34	26	13.0	14.2	0.88	3.94	1	1	0	0	0	0	0	0	0	0	0	0	0	0	head and neck	2	1.69
35	73	21.39	47.06	25	13.2	14.2	0.90	3.94	1	1	0	0	0	0	0	0	0	0	0	0	0	0	head and neck	2	1.7
36	63	22.47	58.82	52	12.2	22.5	0.91	3.52	1	1	0	0	0	0	0	0	0	0	0	0	0	0	head and neck	4	1.7
37	71	18.69	57.32	86	13.3	36.3	0.63	3.4	1	1	0	1	0	0	0	0	0	1	0	1	0	0	head and neck	3	1.57
38	61	17.52	42.25	41	9.2	31.3	0.59	3.02	1	1	0	1	0	0	0	1	0	0	0	1	0	0	head and neck	4	1.42
39	62	16.22	43.80	30	9.9	25.8	0.80	3.47	1	1	0	1	0	0	0	1	0	0	0	1	0	0	head and neck	4	1.37
40	72	23.96	46.51	79	14.8	46.7	0.66	3.97	1	0	0	0	0	0	0	1	0	1	0	1	0	0	head and neck	3	1.72
41	61	19.05	48.19	90	15.5	22.0	0.80	4	1	1	0	0	0	0	0	0	0	1	0	0	0	0	head and neck	4	1.66
42	63	15.93	44.52	54	14.9	23.9	0.61	4.14	1	.	0	0	0	0	0	0	0	0	0	0	0	0	esophageal	4	1.46
43	65	19.33	60.00	54	13.8	15.0	0.51	4.32	0	1	0	1	0	0	0	0	0	0	0	1	0	1	head and neck	4	1.34
44	79	23.12	44.59	46	13.5	17.7	0.98	3.69	1	1	0	1	0	0	0	0	0	0	0	0	0	0	head and neck	4	1.57
45	72	18.85	47.90	48	10.7	17.4	0.89	3.79	1	0	0	0	0	0	0	0	0	0	0	0	0	0	head and neck	4	1.67
46	72	17.74	33.95	50	7.7	26.2	0.84	3.09	1	0	0	1	0	0	0	0	0	0	0	0	0	0	head and neck	4	1.62
47	73	22.39	59.75	56	16.3	20.4	0.75	3.91	1	1	0	0	0	0	0	0	0	0	0	0	0	0	head and neck	4	1.59
48	73	21.84	28.66	75	14.4	24.7	0.78	3.91	1	1	0	0	0	0	0	0	0	0	0	0	0	0	head and neck	4	1.57
49	52	17.44	59.52	92	14.8	22.7	0.55	4.42	1	1	0	0	0	0	0	1	0	0	0	0	0	0	head and neck	3	1.68
50	63	20.63	59.60	59	11.3	23.3	0.65	4.57	0	0	0	0	0	0	0	0	0	0	0	0	0	0	esophageal	4	1.51
51	64	20.39	46.36	62	10.5	23.6	0.79	4.13	0	0	0	0	0	0	0	0	0	0	0	0	0	0	esophageal	4	1.51
52	65	26.96	48.48	42	13.3	16.3	0.65	3.97	1	1	0	1	1	1	0	0	0	0	0	1	0	0	head and neck	4	1.65
53	64	15.14	59.21	67	11.1	34.4	0.60	2.66	1	1	0	0	1	0	0	0	0	0	0	0	0	0	esophageal	4	1.52
54	53	19.57	60.44	53	14.1	25.1	0.63	3.24	1	1	0	0	0	0	0	0	0	0	0	0	0	0	head and neck	4	1.82
55	44	20.44	59.17	81	12.0	19.3	0.87	4.52	1	1	0	0	0	0	0	0	1	0	0	0	0	0	head and neck	4	1.69
56	49	18.81	50.00	59	11.5	27.6	0.67	3.55	1	0	0	1	0	0	1	0	1	0	0	1	1	1	esophageal	4	1.4
57	48	22.02	60.51	74	13.2	21.1	0.61	4.45	1	0	0	0	0	0	0	0	0	0	0	0	0	0	head and neck	2	1.57
58	65	17.38	44.44	41	10.5	18.9	0.34	3.08	0	0	1	1	0	0	1	0	0	0	0	0	0	0	head and neck	3	1.35
59	65	16.93	37.31	67	12.5	17.3	0.46	3.76	0	0	1	1	0	0	1	0	0	0	0	0	0	0	head and neck	3	1.34
60	54	22.92	56.70	64	16.2	19.0	1.01	4.24	1	1	0	0	0	0	0	0	0	0	0	0	0	0	head and neck	4	1.94
61	70	21.85	57.97	47	11.7	9.0	0.49	3.4	0	0	1	1	0	0	0	1	0	0	0	1	1	0	head and neck	4	1.38
62	53	20.99	56.50	75	14.7	30.6	0.79	3.9	1	1	0	1	0	0	0	1	0	0	0	1	0	0	head and neck	4	1.77
63	73	22.27	49.65	47	12.4	22.3	0.60	3.94	0	1	0	0	0	0	0	1	0	0	0	0	0	0	head and neck	3	1.41
64	78	22.70	48.39	161	11.6	11.7	0.58	2.16	1	1	0	1	1	0	0	1	1	0	0	1	0	0	head and neck	4	1.55
65	66	18.84	59.52	94	13.9	27.0	0.96	4.22	1	1	0	0	0	0	0	1	0	0	0	0	0	0	head and neck	4	1.68
66	63	23.13	58.14	68	15.1	22.9	0.86	4.3	1	1	0	1	1	0	1	0	0	0	0	1	0	0	head and neck	4	1.72

67	60	16.74	60.00	74	14.4	24.8	0.53	3.56	1	1	0	0	0	0	0	1	0	0	0	0	0	0	head and neck	4	1.5
68	61	17.50	58.82	31	11.5	27.0	0.81	3.02	1	1	0	0	0	0	0	1	0	0	0	0	0	0	head and neck	4	1.53
69	64	20.87	59.88	81	14.7	26.4	0.82	4.42	1	1	0	1	0	1	0	1	0	0	0	1	1	0	head and neck	4	1.67
70	63	24.09	58.82	59	15.0	26.5	0.72	4.16	1	0	0	1	0	0	0	0	0	0	0	0	1	0	esophageal	4	1.7
71	62	19.30	60.81	49	13.9	19.3	0.90	4.23	1	1	0	0	0	0	1	0	0	0	0	0	0	0	head and neck	4	1.48
72	44	17.86	47.77	67	14.3	25.1	0.57	4.02	1	1	0	0	0	0	0	1	0	0	0	0	0	0	head and neck	4	1.57
73	63	21.36	62.11	84	14.9	33.9	0.91	4.01	1	1	0	1	0	0	0	1	0	0	0	1	1	1	head and neck	4	1.61
74	76	18.50	54.88	50	14.1	12.0	0.84	4.82	1	1	1	1	1	0	1	0	0	0	0	1	1	0	head and neck	4	1.64
75	62	17.27	56.96	41	12.8	18.6	0.81	3.69	1	0	0	0	0	0	0	0	0	0	0	0	0	0	head and neck	3	1.58
76	55	24.15	59.46	54	20.9	17.2	0.69	3.96	1	1	0	1	0	0	0	1	0	0	0	1	1	0	head and neck	4	1.85
77	65	23.50	59.88	50	14.2	19.6	0.62	4.23	1	1	0	1	0	0	0	0	0	0	0	1	0	0	head and neck	2	1.67
78	60	16.79	58.44	61	11.7	28.5	0.87	3.85	1	1	0	0	0	0	0	1	0	0	0	0	0	0	head and neck	4	1.54
79	60	17.70	47.47	36	10.0	25.7	0.85	3.45	1	1	0	0	0	0	0	0	0	0	0	0	0	0	head and neck	4	1.58
80	55	22.59	58.48	52	13.5	19.9	0.78	4.05	1	1	0	0	0	0	0	0	0	0	0	0	0	0	head and neck	3	1.71
81	55	23.43	57.47	37	12.0	23.6	0.95	3.82	1	1	0	0	0	0	0	0	0	0	0	0	0	0	head and neck	3	1.74
82	41	16.95	59.21	91	13.7	33.4	0.60	4.04	1	1	0	0	0	0	0	1	0	0	0	0	0	0	head and neck	4	1.52
83	54	16.91	58.14	90	9.4	28.8	0.67	3.46	1	1	0	0	0	0	0	1	0	0	0	0	0	0	head and neck	4	1.29
84	69	22.28	55.17	79	12.7	30.1	0.51	3.59	1	1	0	0	0	0	0	0	0	0	0	0	0	0	head and neck	4	1.45
85	69	22.15	44.83	74	11.2	40.5	0.55	3.36	1	1	0	0	0	0	0	0	0	1	0	0	0	0	head and neck	4	1.45
86	64	19.03	61.35	157	9.0	68.5	0.73	2.42	1	1	0	0	0	0	0	1	0	1	0	0	0	0	head and neck	4	1.63
87	65	16.96	61.29	108	10.0	39.8	0.57	2.41	1	1	0	0	0	0	0	0	1	0	0	0	0	0	head and neck	4	1.55
